# The quality of care index for low back pain: a systematic analysis of the global burden of disease study 1990–2017

**DOI:** 10.1186/s13690-023-01183-3

**Published:** 2023-09-12

**Authors:** Mohammad Ghafouri, Erfan Ghasemi, Mohsen Rostami, Mahtab Rouhifard, Negar Rezaei, Maryam Nasserinejad, Khashayar Danandeh, Amin Nakhostin-Ansari, Ali Ghanbari, Alireza Borghei, Ali Ahmadzadeh Amiri, Azin Teymourzadeh, Jeffrey B. Taylor, Navid Moghadam, Ramin Kordi

**Affiliations:** 1 Sports Medicine Research Center, Neuroscience Institute, Tehran University of Medical Sciences, Tehran, Iran; 2 Non-Communicable Diseases Research Center, Endocrinology and Metabolism Population Sciences Institute, Tehran University of Medical Sciences, Tehran, Iran; 3 Endocrinology and Metabolism Research Center, Endocrinology and Metabolism Clinical Sciences Institute, Tehran University of Medical Sciences, Tehran, Iran; 4 Department of Neurosurgery, Rush University Medical Center, Chicago, IL USA; 5 Imam Khomeini Hospital Complex, Tehran University of Medical Sciences, Tehran, Iran; 6 Department of Physical Therapy, High Point University, 833 Montlieu Ave., High Point, NC 27262 USA; 7 Spine Center of Excellence, Yas Hospital Complex, Tehran University of Medical Sciences, Tehran, Iran

**Keywords:** Low back pain, Epidemiology, Burden, DALY, YLD

## Abstract

**Background:**

. Low back pain is one of the major causes of morbidity worldwide. Studies on low back pain quality of care are limited. This study aimed to evaluate the quality of care of low back pain worldwide and compare gender, age, and socioeconomic groups.

**Methods:**

. This study used GBD data from 1990 to 2017 from the Institute for Health Metrics and Evaluation (IHME) website. Extracted data included low back pain incidence, prevalence, disability-adjusted life years (DALYs), and years lived with disability (YLDs). DALYs to prevalence ratio and prevalence to incidence ratio were calculated and used in the principal component analysis (PCA) to make a proxy of the quality-of-care index (QCI). Age groups, genders, and countries with different socioeconomic statuses regarding low back pain care quality from 1990 to 2017 were compared.

**Results:**

The proxy of QCI showed a slight decrease from 36.44 in 1990 to 35.20 in 2017. High- and upper-middle-income countries showed a decrease in the quality of care from 43.17 to 41.57 and from 36.37 to 36.00, respectively, from 1990 to 2017. On the other hand, low and low-middle-income countries improved, from a proxy of QCI of 20.99 to 27.89 and 27.74 to 29.36, respectively.

**Conclusion:**

. Despite improvements in the quality of care for low back pain in low and lower-middle-income countries between 1990 and 2017, there is still a large gap between these countries and higher-income countries. Continued steps must be taken to reduce healthcare barriers in these countries.

## Introduction

Low back pain is one of the common causes of morbidity worldwide, with a one-month prevalence of 23.2% [1]. The point prevalence of low back pain has decreased in recent years from 8.2% in 1990 to 7.5% in 2017; however, the number of people with low back pain worldwide has increased from 377.5 million in 1990 to 577 million in 2020 [2]. Low back pain has been the leading cause of years lived with disability (YLDs) from 1990 (42.5 million YLD, 95% Uncertainty Interval (UI) 30.2 to 57.2) to 2017 (64.95 million YLDs, 95% UI 46.5 to 87.4) [2, 3]. The highest incidence of low back pain is in people in their third decade of age [4], and the prevalence of low back pain increases with age, with the highest prevalence in adults aged 80–89 years [2]. There are discrepancies in studies regarding the prevalence of low back pain in males and females. In a systematic review, Fatoye et al. found that in most studies, the prevalence of low back pain has been higher among males [5]; however, based on the global burden of diseases (GBD) project’s data, the point prevalence of low back pain has been higher in females throughout the years [2].

Although low back pain is a major public health issue for people with different demographic characteristics, disparities exist regarding people’s care for low back pain. Taylor et al. reported differences exist between the care people receive for low back pain based on gender and ethnicity [6]. For example, they found that physicians are more likely to suggest surgical treatment or order imaging evaluations for white subjects than black subjects. There were also differences between genders regarding the recommendation of surgical treatment and imaging evaluations [6]. As all patients need appropriate care and services for the treatment of pain, such disparities in the care they receive, which are partly due to their gender and ethnicity, may lead to major health problems globally.

Many health system quality studies consider access to medical services as an indicator of the quality of care. On the other hand, there is a consensus that over-treatment of low back pain has led to worse care [7–9]. In light of the over-treatment paradox, it is clear that access to high-end medical facilities and services cannot be the only index in assessing low back pain care. Quality of care metrics based on public health metrics like prevalence, incidence, and disease burden can help assess the quality of care based on a condition’s actual status instead of merely access indices. GBD health metrics can be incorporated into one score through a principal component analysis, named the Quality of Care Index (QCI), which can help determine care quality and inequality among age and gender subgroups [10–12]. QCI has been used to evaluate the quality of care for several chronic and potentially fatal diseases, including thyroid cancers, brain cancers, and hematological cancers [10–12].

While there are many studies on the consequences and cost-effectiveness of each treatment option for low back pain [8], there is a scarce number of studies on the quality and equality of care for low back pain in geographical, gender, and age categories. In addition, while the quality of care studies are feasible in developed countries because of registries [13–15], the need for registries in developing countries prevents comparing different health systems in low back pain care. Therefore, in our sight of view defining an Index that could demonstrate the disparity of low back pain care across different locations and genders would be extremely valuable in setting future goals. To the best of our knowledge, although there were recent studies about the burden of low back pain in GBD [16, 17], this is the first study aimed to determine the global quality and equality of low back pain care in various geographical, gender, and age categories using GBD data and the newly designed QCI.

## Methods

### Overview and data resources

This study used GBD data from 1990 to 2017 from the Institute for Health Metrics and Evaluation (IHME) website, which employs estimation models for input data from a wide range of resources. IHME provides GBD data using a systematic strategy to explain epidemiologic data on various diseases and risk factors stratified by sex, age, and geographic areas in different categories of nations and regions [3, 18]. The low back pain data is available on the GBD-compare webpage under the GBD code “B.11.3” in the “Causes” section. IHME defines low back pain as “pain in the area on the posterior aspect of the body from the lower margin of the twelfth ribs to the lower gluteal folds (with or without pain referred into one or both lower limbs) that lasts for at least one day. “ GBD is fully aligned with the Guidelines for Accurate and Transparent Health Estimates Reporting (GATHER) Statement, which aims to improve the accuracy and quality of population health research [19]. A review of the methodological design behind the GBD results is outside the scope of our work and has been reported previously and is accessible on the website [18].

### A Proxy of quality of care index

This descriptive study uses GBD metrics to report the current status of low back pain and assess the quality and equality of low back pain care across geographic, gender, and age categories.

Disability-adjusted life years (DALYs) is defined as the sum of years of life lost to early death (YLLs) and YLDs. As no mortality has been reported for low back pain, YLL is negligible, and DALYs and YLD can be used interchangeably in the case of low back pain [20]. YLD = P*DW, where P = prevalence, DW = disability weight of specific disease. So YLD/prevalence = DW is independent of prevalence, and so is DALYs/prevalence. Therefore, DALYs/prevalence shows the burden of low back pain regardless of its prevalence.

Two secondary indices were generated to assess the quality-of-care parameters in this study.

1. DALYs to Prevalence Ratio = DALYs/prevalence.
2. Prevalence to Incidence Ratio = prevalence/incidence.

It was assumed that increases in both measures might show deterioration in care for low back pain. It can be argued that in an episodic and self-limiting condition such as low back pain, the success of care can be defined as shortening episodes, preventing the disease from becoming chronic, and reducing recurrence. The prevalence/incidence would show how long a new low back pain episode will likely last cumulatively until the next year.

These two metrics (prevalence/incidence and DALYs/prevalence) were then combined in a principal component analysis (PCA) to obtain a single score for quality of care, named a proxy of QCI. It should also be noted that we used age-standardized values to calculate the metrics prior to calculating the proxy of QCI. The QCI was calculated beforehand and used in some GBD studies to show care quality and equality [10–12]. As QCI for low back pain due to zero death rate lacked one secondary index in calculations, it was called a proxy of QCI instead of the QCI.

Low back pain care in high-income, high middle income, lower middle income, and low-income countries (based on world bank classifications) [21] and in the age categories of 15–59 years and over 60 years were compared. The classification of countries was based on the World Bank country reports on income levels. Based on previous studies showing that the prevalence of low back pain gradually increases from the teenage years to 60 years of age and then decreases, we chose the categories 15–59 and over 60 years old to assess the impact of age on the index of care [22].

### Gender disparity ratio

To yield insight into the disparity of quality of care services between males and females with LBP, the gender disparity ratio (GDR) was used. It was calculated by dividing males’ proxy of QCI by females’ QCI across countries. A GDR equal to 1 indicates no gender disparity. However, values lower or higher than one was indicative of disparity against males and females, respectively.

### Validation of the Proxy of QCI

To validate the proxy of QCI, a mixed model was used to compare it to the Healthcare Access and Quality Index (HAQI) [23]. In this model, proxy of QCI was set as the dependent variable, and inpatient and outpatient health care utilization, DALY, and prevalence of LBP were set as independent parameters, with different nations considered as random effects parameters of the model. The Pearson’s correlation coefficient between the predicted values with the HAQI was 0.77, indicating an acceptable correlation [24].

### Statistical analysis

Based on the GBD study, primary index values were reported with a 95% uncertainty interval (UI) of all age numbers and age-standardized rates per 100,000 population, and if the UIs of two strata did not overlap, shifting trends were regarded as significant [25]. For the analysis, age-standardized indices were used to compare countries with different age structures. Six-sigma tests were used to investigate the distribution of outliers in GBD metrics.

The secondary indices were combined in a PCA model to obtain a single score for quality of care measurement. PCA is a multivariable statistical technique that results in uncorrelated components through a linear combination of variables [26]. The first emerged component of PCA carries the most information about input variables. This component was termed a proxy of QCI and created a scale from 0 to 100, with 0 representing low quality and 100 representing the optimal quality of care. The calculated score was then used to compare the proxy of quality of care across geographic, gender, and age categories. Gender equality was assessed by dividing the score of males of each country to the scores of females, any deviation from 1 would indicate gender inequality.

All statistical analyses and plots were performed by R statistical packages v3.6.1 (http://www.r-project.org/, RRID: SCR _001905) [27]. Additional details of the calculation and codes are available in a previously published protocol about the QCI [28].

## Results

### Incidence, prevalence, DALYs, and other epidemiologic indices

The age-standardized point prevalence, incidence, and YLD of LBP in the world by sex in 1990 and 2017 are summarized in Table 1. The results show that in 2017 about 552 (95% UI 491 to 621) million people suffered from LBP worldwide, and the prevalence of LBP was 7013.7 (6247.9 to 7891.9) per 100,000 people in the world. Compared to 1990, 43% more people were affected by low back pain in 2017, but the prevalence rate of low back pain decreased by 16%. There were higher crude incidence and YLD in 2017 than in 1990, but the incidence and YLD rate per 100,000 people decreased by 13.7% and 15.9%, respectively. Furthermore, the prevalence, incidence, and YLD age-standardized rates of LBP in females were higher than in males in both years.

**Table 1:** Global prevalence, incidence, and YLD across countries and age-standardized rates for both sexes, females, and males, in 1990 and 2017 (with 95% uncertainty interval (UI))

	Prevalence		Incidence		DALYs (YLD)	
	Cases (million)	Rate (per 100,000)	Cases (million)	Rate (per 100,000)	YLD (million)	Rate (per 100,000)
1990						
Both	386 (343 to 434)	8341.1 (7389.8 to 9370.1)	149 (131 to169)	3168.9 (2799.7 to 3572.9)	43 (31 to 58)	932.5 (658.5 to 1248.3)
Female	224 (199 to 253)	9528.3 (8455.2 to 10736.5)	85 (75 to 96)	3550.6 (3141.5 to 4008.1)	25 (33 to 18)	1059 (748.5 to 1414.0)
Male	161 (143 to 181)	7085.9 (6299.5 to 7939.4)	64 (56 to 73)	2770.2 (2447.1 to 3131.8)	18 (13 to 24)	799.3 (562.0 to 1069.5)
2017						
Both	552 (491 to 621)	7013.7 (6247.9 to 7891.9)	216 (191 to 244)	2748.9 (2428.7 to 3101.6)	62 (43 to 83)	785.1 (554.3 to 1051.5)
Female	320 (285 to 360)	7949.9 (7084 to 8961.1)	124 (110 to 139)	3086.9 (2735.7 to 3476.8)	35(25 to 48)	884.4 (626.1 to 1184.2)
Male	232 (206 to 260)	6022.4 (5362.9 to 6727.9)	92 (81 to 105)	2392.94 (2109.3 to 2703.9)	26 (18 to 35)	680.3 (478.9 to 909.9)

**Fig. 1:**
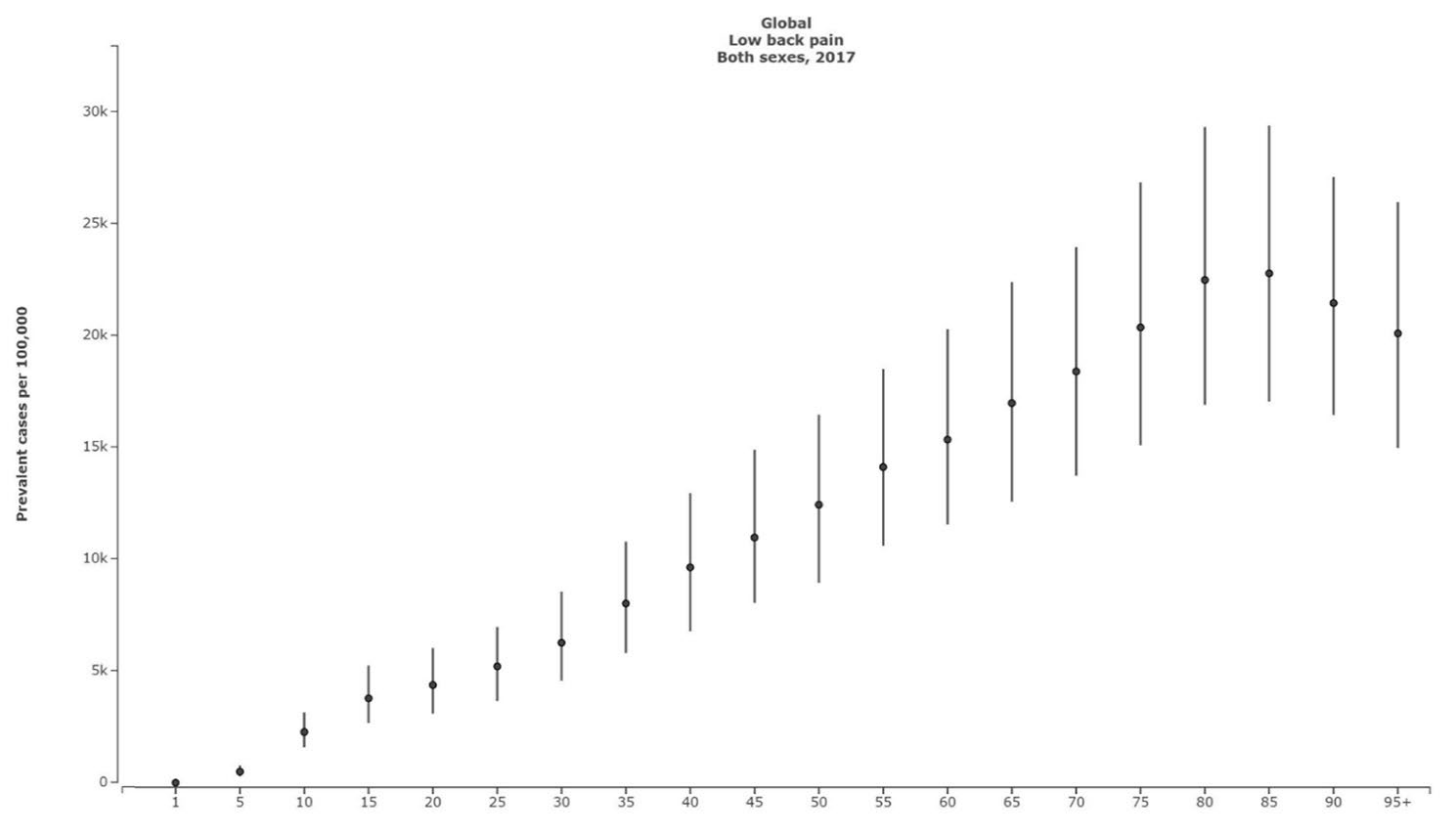
The global point prevalence rate with 95% uncertainty intervals in different ages in 2017

When comparing different age groups, the result showed that after a steady increase, the prevalence cases of LBP peaked in the age group of 85–89 years with 22,786.71 (17,047.44 to 29,402.05) cases per 100,000. This pattern was observed in both females and males in 2017. YLD and incidence rates also follow the same pattern, peaking in the 85–89 years age group for both sexes (Fig. 1).

**Fig. 2:**
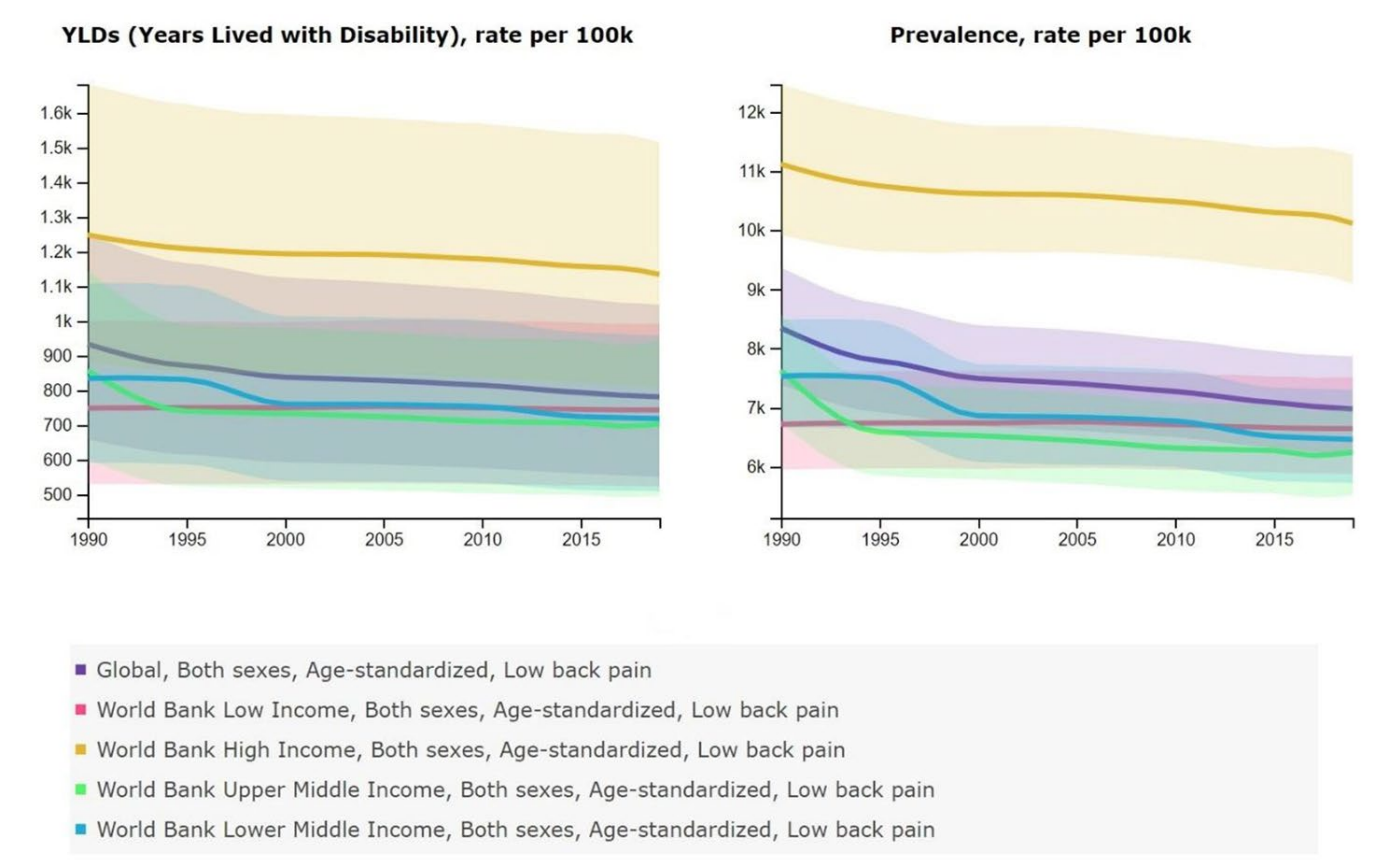
Age-standardized YLD and Prevalence of low back pain, 1990–2017, in global level, in different income level countries

When comparing YLD and the prevalence of LBP between countries with different income levels, the results show the lower YLD and prevalence rate in 2017 compared to 1990 in all income levels. High-income level countries represent the highest YLD and prevalence rate of low back pain in 1990 and 2017. In 2017, The YLD and prevalence rate of high-income countries were 1,151.6 (816.6 to 1,538.5) and 10,261.31 (9,259.0 to 11,408.7) per 100,000 persons, respectively. In addition, upper-middle-income countries had the lowest YLD and prevalence rates in 2017 at 696.24 (491.16 to 930.20) and 6,186.76 (5,478.93 to 6,936.73) per 100,000 persons, respectively (Fig. 2).

### A proxy of the quality of care index

**Fig. 3:**
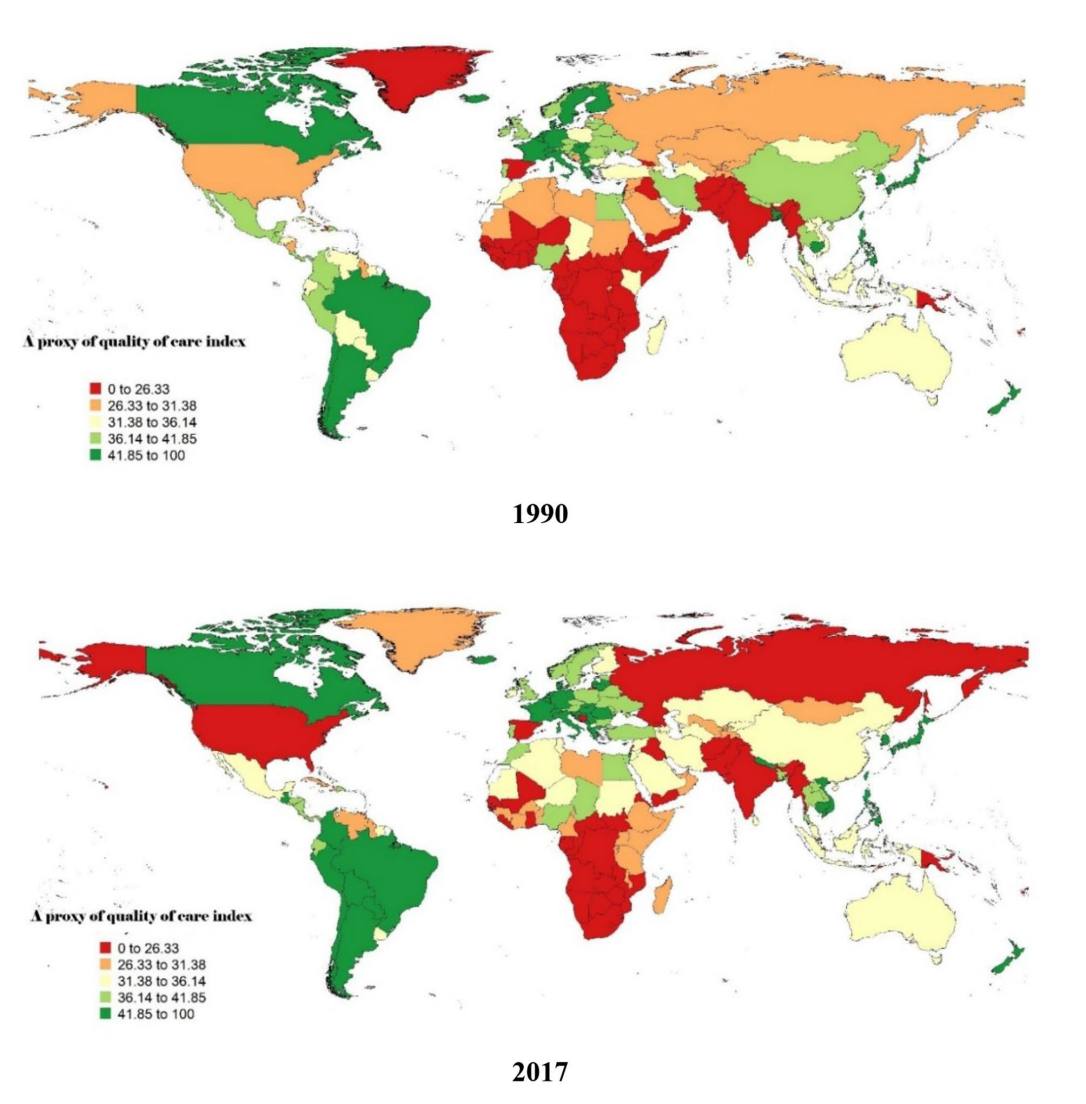
A proxy of quality of care index (QCI) of Low back in 1990 and 2017(age standardized)

The proxy of QCI for low back pain was calculated to compare the quality and equity of low back pain care across countries (Fig. 3). The index showed a slight decrease from 36.44 in 1990 to 35.20 in 2017. High- and upper-middle-income countries showed a decrease in the quality of care from 43.17 to 41.57 and from 36.37 to 36.00, respectively, from 1990 to 2017. On the other hand, the quality of care in low and low-middle-income countries improved, from a proxy of QCI of 20.99 to 27.89 and 27.74 to 29.36, respectively. While the score for the 15–59 age category is better in high-income and upper-middle-income countries than for the over-60s in 2017, this is not the case in low-income and lower-middle-income countries, where the score for the 15–59 age category is lower than for the over-60s (Table 2).

**Table 2:** A proxy of quality of care index in countries with different income levels and two age categories of 15–59 and over 60 in 1990 and 2017

Location	Year	Age category	Sex	DALY/Prevalence Ratio	Prevalence/Incidence Ratio	Low back pain care index
**Global**	1990	Age-standardized	Both	0.11223	2.3657	36.44
	2017			0.11242	2.3416	35.20
**High-income**	1990	Age-standardized	Both	0.11307	2.3745	43.17
	2017			0.11301	2.3635	41.57
**Upper-middle-income**	1990	Age-standardized	Both	0.11241	2.3534	36.37
	2017			0.11278	2.3249	36.00
**Lower-middle-income**	1990	Age-standardized	Both	0.11117	2.3526	27.74
	2017			0.11170	2.3330	29.36
**Low-income**	1990	Age-standardized	Both	0.11058	2.3274	20.99
	2017			0.11150	2.3325	27.89
**High-income**	1990	15–59	Both	0.11478	2.3848	44.48
	2017			0.11478	2.3742	43.43
**Upper-middle-income**	1990	15–59	Both	0.11485	2.3476	41.34
	2017			0.11507	2.3453	42.87
**Lower-middle-income**	1990	15–59	Both	0.11371	2.3399	31.51
	2017			0.11423	2.3286	34.53
**Low-income**	1990	15–59	Both	0.11309	2.3019	22.76
	2017			0.11392	2.3065	29.87
**High-income**	1990	Over 60	Both	0.10342	2.6856	34.09
	2017			0.10342	2.6640	32.65
**Upper-middle-income**	1990	Over 60	Both	0.10208	2.6888	44.07
	2017			0.10257	2.5895	34.04
**Lower-middle-income**	1990	Over 60	Both	0.10068	2.6852	54.13
	2017			0.10113	2.6431	48.11
**Low-income**	1990	Over 60	Both	0.10087	2.6504	50.44
	2017			0.10181	2.6711	44.91

### Gender disparity ratio

**Fig. 4:**
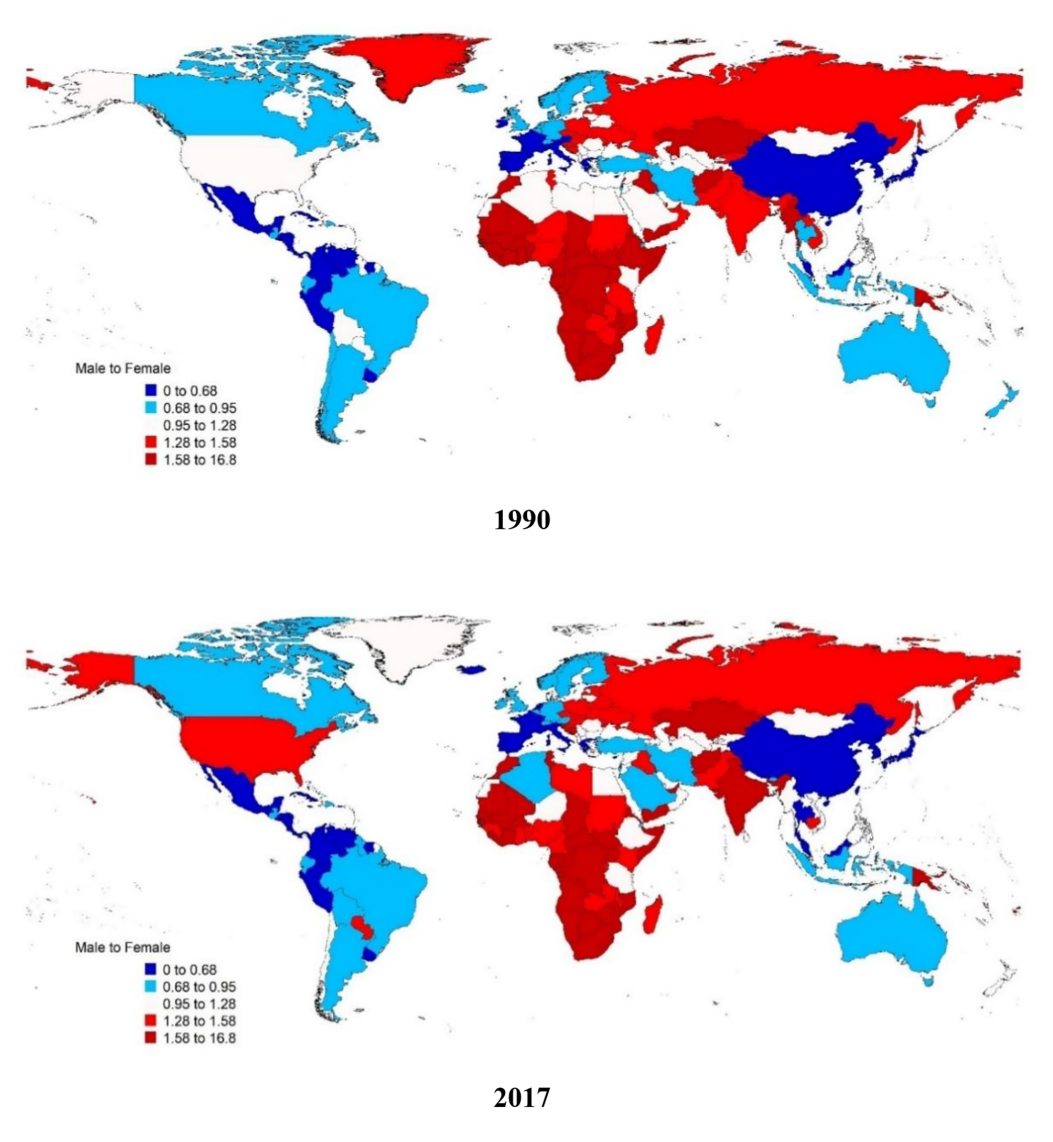
Gender inequity in a proxy of the quality-of-care index (QCI) of Low back pain, 1990, 2017

**Fig. 5:**
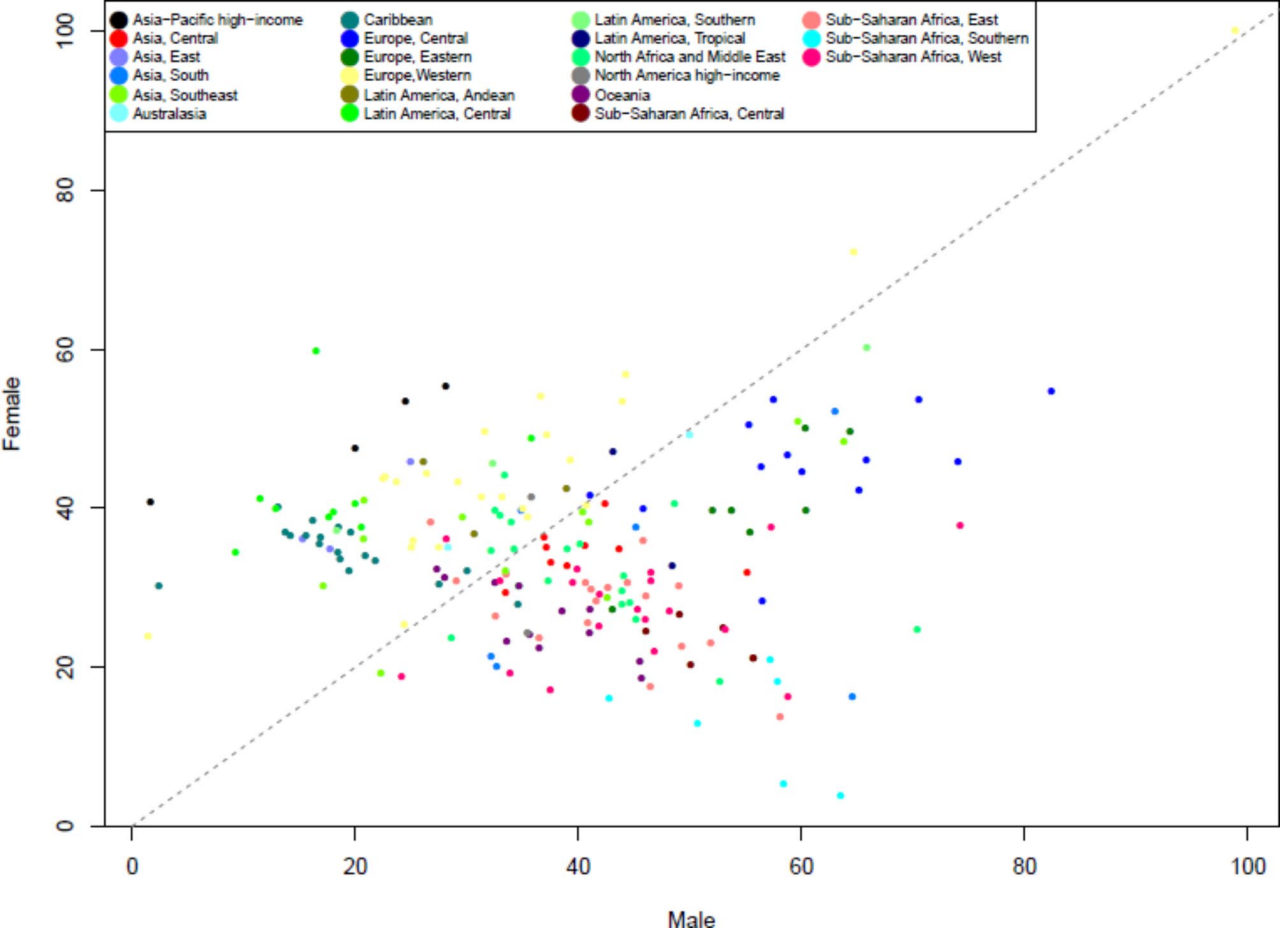
Countries scatter plots of a proxy of quality of care index (QCI) for female and male in 2017

Gender inequality in the quality of care for low back pain is shown in Fig. 4. When comparing gender inequality, GDR decreased from 1.0100 in 1990 to 0.9802 in 2017, indicating that the proxy of QCI for men compared to women was lower in 2017 than in 1990. In addition, GDR in 2017 was lower than 1 in high-income and upper-middle-income countries, which means a higher quality of care for women compared to men, and it was higher than 1 in low-income and lower-middle-income countries, which indicates that a higher quality of care for men compared to women in these countries (Table 3). Furthermore, the scatter plot of QCI based on gender between different regional categories showed no pattern between specific regional categories. (Fig. 5).

**Table 3:** Gender Inequality of the proxy of quality of care index for low back pain in different income level countries in both sexes in 1990 and 2017

Location	Year	Age category	Sex	DALY/Prevalence Ratio	Prevalence/Incidence Ratio	Low back pain care index	Gender disparity ratio
**Global**	1990	Age-standardized	Male	0.11175	2.3295	37.97	1.0100
			Female	0.11288	2.3914	37.59	
**Global**	2017	Age-standardized	Male	0.11298	2.3170	35.34	0.9802
			Female	0.11197	2.3596	36.05	
**World Bank High-Income**	2017	Age-standardized	Male	0.11347	2.3413	34.55	0.8195
			Female	0.11262	2.3791	42.16	
**World Bank Upper-Middle-Income**	2017	Age-standardized	Male	0.11343	2.2944	28.42	0.7653
			Female	0.11231	2.3473	37.13	
**World Bank Lower-Middle-Income**	2017	Age-standardized	Male	0.11242	2.3161	40.02	1.3692
			Female	0.11114	2.3452	29.22	
**World Bank Low-Income**	2017	Age-standardized	Male	0.11105	2.3178	44.46	1.5381
			Female	0.11192	2.3478	28.90	

## Discussion

This is the first study to use GBD data with the newly developed proxy of QCI to assess the overall quality and equity of low back pain treatment across different geographic, gender, and age groups. Volinn et al. suggested that improvement in the indices to compare the quality of low back pain care in different countries was needed, as there are disparities regarding the prevalence of low back pain across countries [29]. In this study, we proposed a new method to compare and analyze the quality of care in different regions called the proxy of QCI. The low back pain GBD crude measures, such as prevalence and YLDs, were reported in a similar publication. Still in this study, we used these indicators to calculate secondary indices to highlight quality and equity of care in different areas and genders, making it more practical for policymakers [16, 17].

Despite the increase in the crude prevalence, incidence, and YLD in the results, this study showed that the prevalence, incidence, and YLD rate of low back pain per 100,000 individuals decreased between 1990 and 2017. The growth of the population from 1990 to 2017, from 5.33 to 7.55 billion, can be the main cause of the total rise in prevalence, incidence, and YLD [30]. Furthermore, the global median age has increased in recent decades [31]. Evidence also indicates that a history of low back pain is the single most significant predictor of future low back pain, suggesting that older adults have a greater chance of developing subsequent low back pain [32]. On the other hand, the lower prevalence and YLD rate per 100,000 might show an improvement in the quality of care and medical management through the disease between 1990 and 2017. Also, the lower incidence rate can be due to lowering risk factors. For example, globally, the percentage of the population that smokes every day has decreased, which may play a role in decreasing the incidence and prevalence of low back pain, considering smoking as a risk factor for low back pain [33, 34]. Also, the higher quality of life and education due to globalization in recent decades can play an important role in decreasing the incidence rate of low back pain [35].

Results indicate that the female gender had the higher prevalence, incidence, and YLD age-standardized rate across years. In previous studies, it was shown that females are more prone to low back pain due to their higher pain sensitivity, lower lumber muscle mass, and typical changes in their lifecycle, including pregnancy, childbirth, hormonal imbalances, and menopause, which may all have a role in higher low back pain rates in females compared to males [36, 37].

Results showed that low back pain prevalence peaked at the 85–89 age group in 2017. The same pattern was observed for both sexes and incidence and YLD rates. The results are similar to previous studies, which showed that low back pain becomes more common with advancing age [22]. This may be attributed to low physical activity, osteoarthritis, spinal stenosis, and degeneration of joints in the lumbar spine [38, 39]. However, some evidence declined this relation [40].

Lower global prevalence and YLD rate of low back pain per 100,000 individuals with different socioeconomic statuses in 2017 compared to 1990 were shown in our results. As the proxy of QCI showed, it may be mainly due to the improvement of low-income and lower-middle-income countries in the quality of care for back pain and higher global educational level and lower poverty rates, which both boost health care among people [41]. However, the highest prevalence and YLD were among high-level income countries in 1990 and 2017, possibly due to better patient data registration and higher quality data in these countries. Also, it has been shown that people with worse socioeconomic status might be less sensitive to pain and might not seek care for less severe pain, which can lead to underestimation of the low back pain cases in countries with poor socioeconomic status [42].

Proxy of QCI showed a slight global decrease in the quality of care for low back pain from 36.44 in 1990 to 35.20 in 2017. While the low-income and lower-middle-income countries have improved in terms of quality of care for back pain, the high-income and higher-middle-income countries had lower quality of care in 2017 compared to 1990. Furthermore, it was shown that the highest quality of care in high-income and higher-middle-income countries was dedicated to those aged 15–59 years as opposed to lower and lower-middle-income countries, where people aged 60 years or older had the highest quality of care for low back pain. Older people have the highest prevalence and YLD, so this difference can explain why the quality of care for low back pain has decreased in high and higher-middle-income countries where older adults receive worse care than younger adults. Despite the improvement in low-income and low-middle-income countries in QCI, there is still a considerable difference compared to higher-income countries. It is documented that more healthcare expenditures lead to better healthcare. The low-income countries have the lowest expenditures, which can explain the difference between these countries and high-income countries in QCI [43]. The WHO reports between 2000 and 2017 showed that the health spending of low and middle-income countries increased annually by 6.3 and 7.8%, respectively, which may explain the increase in quality of care in these countries in 2017 compared to 1990 [44].

We also found out that while the global male-to-female quality of care has changed in favor of the female gender since 1990, it is not the case for all countries, and low and lower-middle-income countries show a male-to-female gap in favor of the male gender. Previous studies showed that women in low and lower-middle-income countries have more barriers to healthcare. Also, they have lower access to resources such as education, employment, and money, which are the main reasons for these societies undervalue women and prioritize men’s health [45, 46]. There is a need for interventions to increase awareness of gender norms and empower women to advocate for their health in these countries [47].

### Limitations

There were no data regarding ethnicity in the GBD database, which is the main limitation of our study as there may be considerable differences in the quality of low back pain across ethnicities. Also, the heterogeneity and accuracy of data collection and reporting across countries may affect the results.

## Conclusion

In this study, we introduced scale to examine the quality of care for low back pain in various regions, as well as the disparities in low back healthcare between men and women. Despite an increase in the quality of care for low back pain in low and lower-middle-income nations between 1990 and 2017, there is still a large gap between these countries and higher-income countries, which must take initiatives to enhance healthcare in these countries.

## Data Availability

The data that support the findings of this study are openly available in the Institute for Health Metrics and Evaluation (IHME) website at [https://gbd2017.healthdata.org/gbd-compare/] [48].
